# Structure relaxation and crystallization of the CoW-CoNiW-NiW electrodeposited alloys

**DOI:** 10.1186/1556-276X-9-66

**Published:** 2014-02-10

**Authors:** Evgeny V Pustovalov, Evgeny B Modin, Oleg V Voitenko, Aleksander N Fedorets, Aleksander V Dubinets, Boris N Grudin, Vladimir S Plotnikov, Sergey S Grabchikov

**Affiliations:** 1 Far Eastern Federal University, Shukhanova 8, Vladivostok 690950, Russia; 2 Scientific and Practical Centre of Material Science, Belarus National Academy of Sciences, P. Brovki 19, Minsk 220072, Belarus

**Keywords:** Electron microscopy, Nanocrystalline CoW-CoNiW-NiW alloys, Crystal growth, *In situ* experiments, HAADF STEM, EELS, EDS, 68.37.Ma, 61.46.-w, 79.20 Uv

## Abstract

The structure of electrolytically deposited nanocrystalline alloys of the CoW-CoNiW-NiW systems under low-temperature heating was investigated by means of high-resolution transmission electron microscopy (HRTEM), high-angle annular dark-field scanning transmission electron microscopy (HAADF STEM), and analytical methods such as energy dispersive x-ray spectroscopy (EDS) and electron energy loss spectroscopy (EELS). Structural relaxation and crystallization were investigated at temperatures of 200°C to 300°C. The structural and compositional inhomogeneities were found in the CoW-CoNiW-NiW alloys, while the local changes in composition were found to reach 18 at.%. Nanocrystals in the alloys grew most intensely in the presence of a free surface, and we found their nuclei density to range from 2 × 10^23^ /m^3^ to 3 × 10^23^ /m^3^. It was determined that the local diffusion coefficient ranged from 0.9 to 1.7 10^−18^ m^2^/s, which could be explained by the prevalence of surface diffusion. The data gathered in these investigations can be used to predict the thermal stability of CoW-CoNiW-NiW alloys.

## Background

Tungsten-based alloys with iron group metals (Ni and Co), particularly CoW and CoNiW, possess better functional properties and in our case alloys were formed by electrochemical deposition. These alloys can be used as thermo-resistant and hard-wearing materials [1,2] and as alternatives to chromium coatings [3]. Tungsten-based alloys can be found in hydrogen power engineering, sewage sterilization, and toxic waste putrefaction [4]. Thin magnetic films based on CoNiW alloys are promising as materials for perpendicular or near-perpendicular magnetic recording because of their columnar structure with perpendicular magnetic anisotropy [5-7]. Researchers are interested in these films because of their wide range of magnetic properties that are dependent on deposition conditions and chemical composition [4-6,8-10]. It is well known that the alloy structure of CoW-CoNiW-NiW may be nanocrystalline or amorphous depending on the composition and preparation conditions [7-14]. At the same time, the degree of order of the structure significantly changes depending on the processing history of the alloy. One simple treatment, low-temperature annealing, is interesting from a practical perspective. While the structure changes of these alloys are well-studied at higher temperatures, they are not well-studied between 200°C and 300°C. However, the initial stages of atomic structure relaxation and crystallization are extremely important in order to understand further changes in the macrostructure and physical properties.

## Methods

Deposition was performed in stationary- and pulsed-current conditions at frequencies of 1 to 10 kHz. A 0.1-mm-thick polished copper foil was used as the substrate. Studies of the microstructure were performed on films 40- to 80-nm thick, placed on standard copper grids for transmission electron microscopy (TEM). *In situ* heating experiments were used according to various schemes. In one case, heat was applied at a constant rate of 1 to 2°С/min to a maximum temperature of 300°C. In another, it was applied stepwise in increments of 50°С. Isothermal annealing was performed at 200°C, 250°C, and 300°C. Three electron microscopes were used: FEI Titan™ 80–300 (FEI Company, Hillsboro, OR, USA), JEOL ARM™ 200 (JEOL Ltd., Tokyo, Japan) equipped with aberration correctors of the objective lens, and Carl Zeiss Libra® 200FE (Carl Zeiss AG, Oberkochen, Germany) equipped with an omega filter. Local chemical analysis was completed using both energy dispersive x-ray spectroscopy (EDS) and electron energy loss spectroscopy (EELS). The accelerating voltages were 80 and 300 kV for the Titan, and 200 kV for the ARM200 and Libra 200FE. *In situ* experiments were carried out using the FEI Titan 80–300 and Zeiss Libra 200 FE with a specialized Gatan dual-axis heating holder (Gatan, Pleasanton, CA, USA). Comparable *in situ* heating experiments were carried out with the Libra and Titan, both with and without electron beam irradiation. It was found that electron beam irradiation can lead to a temperature difference in the specimen of up to 300°C, depending on the current density of the electron beam.

## Results and discussion

The CoW-CoNiW-NiW alloys have a quasi-network structure, with nanocrystals in the cells separated by a ‘skeleton’ amorphous structure [11,12]. The high scattering capability of the tungsten atoms allows the ordered structure to be visualized by aberration-free high-resolution transmission electron microscopy (HRTEM) with sufficient contrast down to an area on the order of 1 nm, which is a few unit cells of the crystalline phases of tungsten as well as the crystalline phases and solid solutions of NiW and CoW.

It is well known that a NiW alloy structure changes due to the concentration of tungsten [13]. Below 19.6 at.% W, the structure is crystalline, whereas above 23.5 at.% it is amorphous. If the composition is between these two values, the structure is in a transition zone between crystalline and amorphous. Chen at al. [14] investigated the transition range under low-temperature annealing and found that at 19.6 at.%, W, the as-prepared alloy's structure, was completely crystalline. In that case, the NiW alloy film was prepared by magnetron deposition and was about 1-μm thick. A metastable crystalline phase can form under those conditions. Our NiW alloy film was prepared by electrochemical deposition at a thickness of about 40 to 80 nm. The temperature difference of the surface atoms as well as the tungsten concentration (32 at.% in our case) explain the initial structural differences.

**Figure 1:**
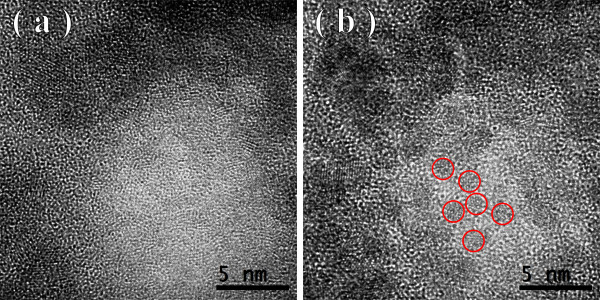
TEM image of NiW alloy: initial state (a) and after heating for 250 s (b).

**Figure 2:**
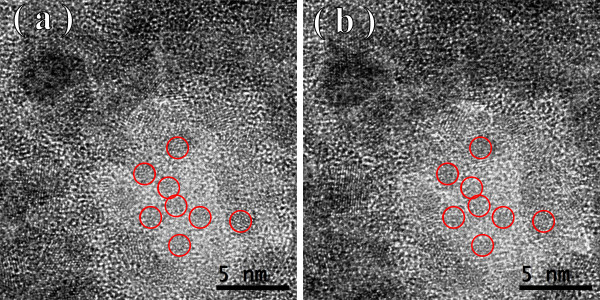
Structure of the NiW alloy after heating for 385 s (a) and 535 s (b).

**Figure 3:**
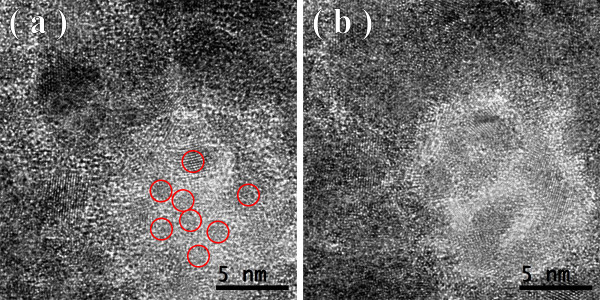
Structure of the NiW alloy after annealing for 800 s (a) and 1,275 s (b).

**Figure 4:**
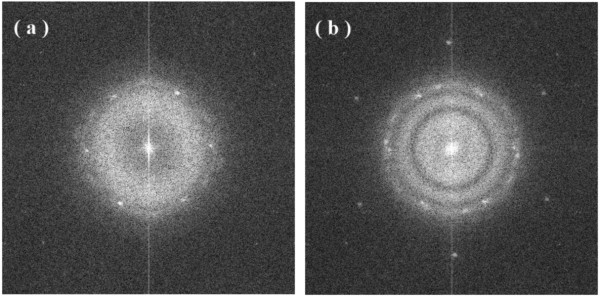
**Fourier spectra of the images for Figure**1**a (a) and Figure**3**b (b).**

Figures 1, 2, 3 show the transmission electron microscopy images of the area of the NiW alloy structure which changes during the heating process at 250°C. Images were taken from the Titan at 80 kV. In the initial state (Figure 1a), only the boundaries of the network show signs of a nanocrystalline structure where the cells have a structure with a low degree of order. In the image, ordering can be seen at the atomic distances of 1 to 2 periods. In the annealing process, in areas with an amorphous structure, nuclei appeared with a high degree of order. After aging for 250 s at a temperature of 250°C, their size was about 1.5 nm (Figure 1b). The density of the nuclei was 2 × 10^23^/m^3^. After aging for 385 s at 250°C, the density increased to 3 × 10^23^/m^3^, but there was almost no change in their mean size (Figure 2a). Their growth began after heating for 1,275 s to an average size of about 4 nm (Figure 3b). At that time, the structure of the nanocrystalline matrix became more ordered. As can be seen from the Fourier spectra in the initial state (Figure 4a), the only reflections visible corresponded to a spatial period of 0.2 nm, whereas after annealing, additional reflections could be seen that corresponded to a spatial period of 0.12 nm (Figure 4b). This indicated an increase in the degree of long-range order in the crystal structure of the matrix.

**Figure 5:**
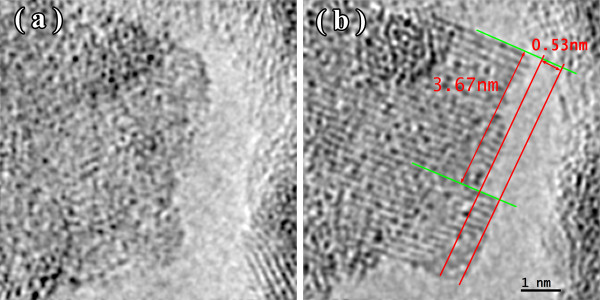
**A nanocrystal in NiW alloy: initial state (a) and at 300°C for 160 s (b).**

**Figure 6:**
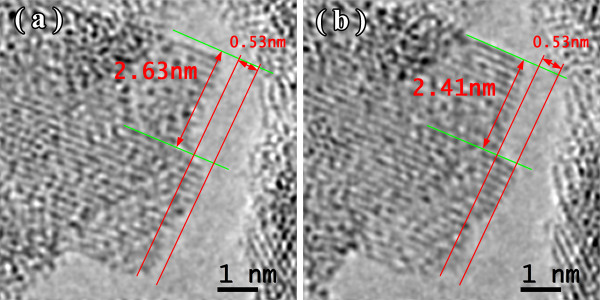
**TEM image of NiW alloy structure at 300°C for 204 (a) and 230 s (b).**

**Figure 7:**
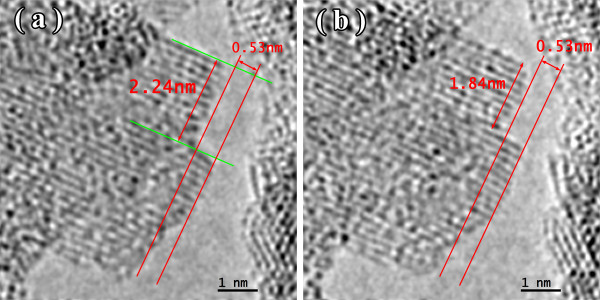
**TEM image of NiW alloy structure at 300°C for 246 (a) and 264 s (b).**

Similar to the CoP alloys [15-17], the most intense growth of nanocrystals in the NiW alloy took place when there was a free surface. In the initial state, at the pore borders, the nanocrystal did not have a high degree of order (Figure 5a), and the Fourier spectrum showed diffuse reflections corresponding to a spatial period of 0.2 nm. After heating for 160 s at 300°C, the nanocrystal structure became more ordered, with smooth boundaries along the matrix (Figure 5b). Upon further heating (Figures 6 and 7), growth occurred mainly at the free surface. An online supplemental video file was provided to see this in more detail (Additional file 1). The overall heating time was 264 s. Images were taken from the Titan at 300 kV.

**Figure 8:**
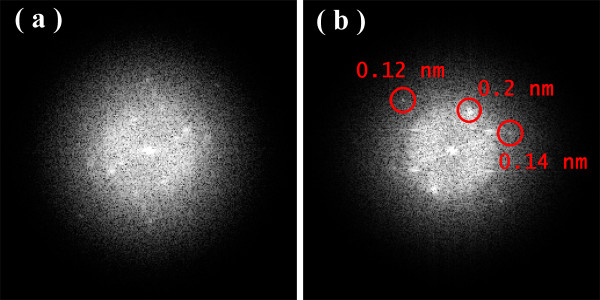
**Fourier spectra of the TEM images Figure**5**a (a) and Figure**6**b (b).**

It should be noted that the NiW nanocrystal growth along the free surface occurred in areas 0.53 nm wide. Analysis of the Fourier spectra from Figure 5a,b showed periods of 0.2, 0.14, and 0.12 nm in the structure of the alloy (Figure 8). This is likely due to β-W (ICSD 52344). Because of the phases for Ni, W, and their combinations, β-W is the only one with the appropriate lattice parameter. We assumed that, on a free surface, growth occurs by increments on one elementary cell. Unfortunately, in this case, the nanocrystal orientation was such that the atomic planes parallel to the free surface could not be seen. Accordingly, the volume of material transferred in 60 s was anywhere from 0.84 to 1.68 nm^3^. The volume of an elementary cell of β-W is 0.12879 nm^3^, meaning that between 6 and 13 elementary cells, 48 to 104 atoms were deposited in 60 s. The coefficient of diffusion ranged from 0.9 to 1.7 × 10^−18^ m^2^/s.

It is well known that the local atomic structure can be modified by an electron beam and is visible in TEM as radiation damage, nanoparticle coagulation, or other changes [18-21]. The density of such areas and the level of structure damage depend on the current density and the incident beam energy. In our investigations, the current density did not exceed 10 to 20 A/cm^2^ at beam energy of 80 to 300 kV. This allowed us to choose the conditions under which local structure modification was negligible and not visible under electron beam irradiation.

One method proposed for estimating diffusion coefficients of amorphous alloys is by direct measurement of the crystals' size changes under heat using the electron microscope [22]. We estimated the diffusion coefficient by direct observation of atoms moving in the specimens by using TEM with high-pass diffusion [23] at the beginning of structure relaxation and at crystallization at elevated temperatures. The most visible changes in the alloy structure occurred at the vacuum-crystal interface. In these areas, the local diffusion coefficient was much higher, up to 10^−18^ cm^2^/s. This does not contradict prior findings that the mean value of the diffusion coefficient ranges from 10^−25^ to 10^−24^ cm^2^/s for Co/Ni in W and W in Co/Ni [24,25] at 200°C. Our primary goal was to estimate the diffusion coefficient through direct local observation of the beginning of atomic structure relaxation and crystallization at low-temperature annealing.

**Figure 9:**
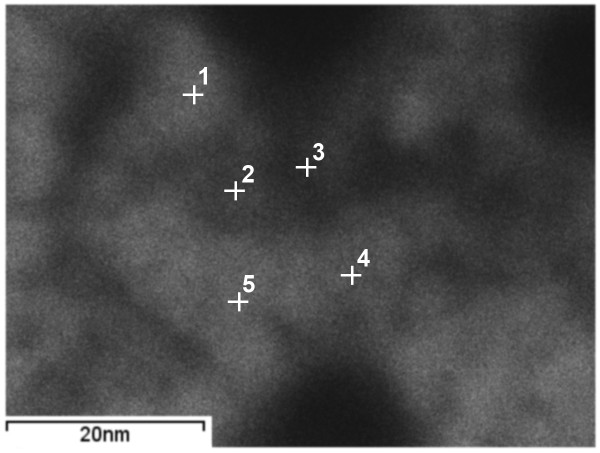
STEM image of the NiW alloy structure with the points of EDS analysis.

**Figure 10:**
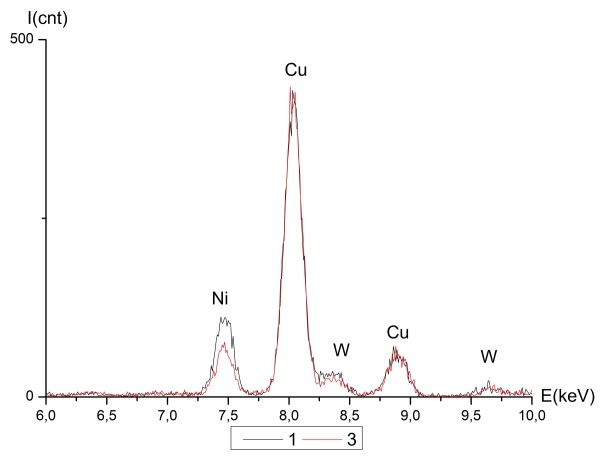
**The EDS spectra of K and L lines of NiW in points 1 and 3 (Figure**9**).**

Investigations of local chemical composition using EELS and EDS showed an inhomogeneous distribution of elements in the NiW alloy. Figure 9 shows the high-angle annular dark-field scanning transmission electron microscopy (HAADF STEM) image of an area with points for analysis. Lighter areas correspond to thicker regions and/or higher average atomic numbers, while the darker areas correspond to thinner regions and/or lower average atomic numbers. Table 1 shows the results of the processed EDS spectra where the W content was higher in thinner areas. The average composition of the thicker areas was 30 ± 0.74 at.% W, whereas the composition of the thinner areas was 34 ± 1.2 at.% W. Figure 10 shows the EDS spectra graphs of K and L lines for points 1 and 3. The presence of Cu, corresponding to the signal from the copper TEM grid supporting the specimen, and oxygen was clearly seen.

**Table 1:** Ni and W content of NiW alloy at the points of interest using EDS analysis

	**Atomic percentage of Ni**	**Atomic percentage of W**
Spectrum 1	70.55	29.45
Spectrum 2	66.73	33.27
Spectrum 3	65.03	34.97
Spectrum 4	70.46	29.54
Spectrum 5	69.23	30.77

**Figure 11:**
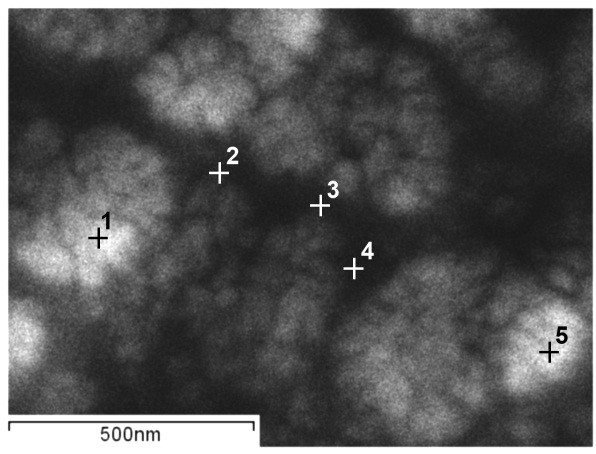
STEM image of the CoW alloy structure with the point for EDS analysis.

**Figure 12:**
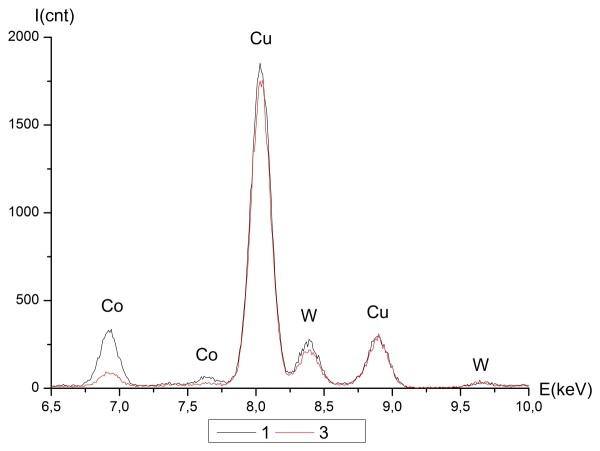
**The EDS spectra of K and L lines of CoW in points 1 and 3 (Figure**11**).**

**Figure 13:**
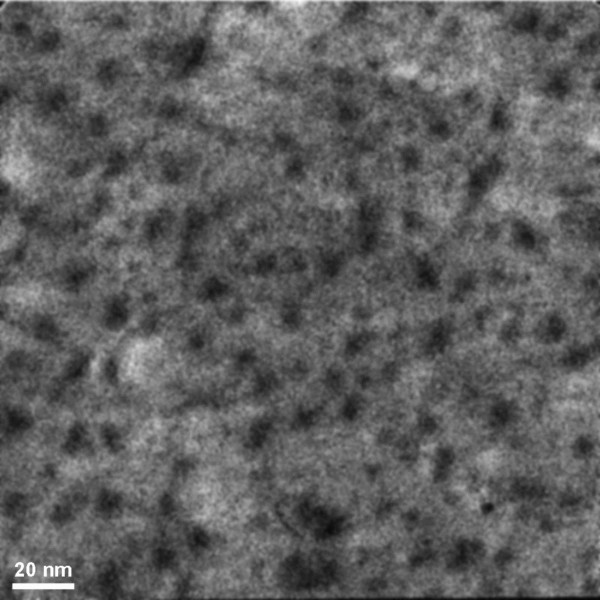
ESI image of the nickel map, taken from the Libra at 200 kV.

**Figure 14:**
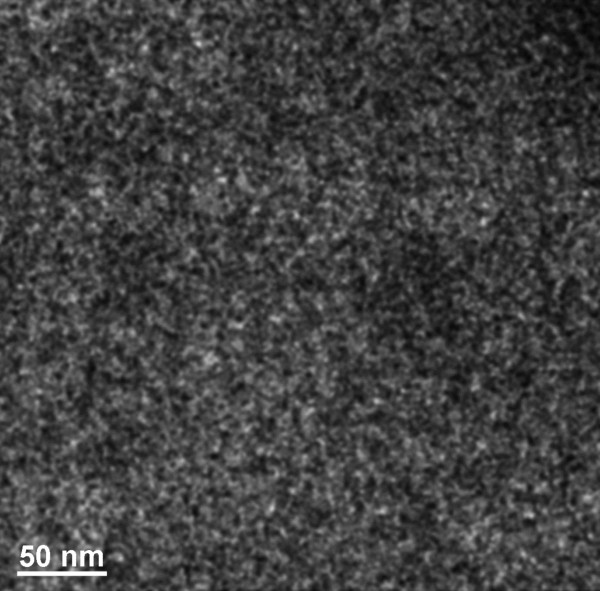
ESI image of the cobalt map, taken from the Libra at 200 kV.

CoW alloy had a similar composition distribution. Figure 11 shows the STEM image of the CoW alloy structure with points for EDS analysis. Table 2 shows the results of the processed EDS spectra. Figure 12 shows the EDS spectra graphs of K and L lines for points 1 and 3. The average composition of the thicker areas was 34 ± 2.6 at.% W, whereas the thinner areas were 52 ± 1.5 at.% W. Electron spectroscopic images (ESI) obtained by EELS for the nickel and cobalt K lines showed the heterogeneous distribution in the alloy structure. Figures 13 and 14 show the images for nickel and cobalt, respectively. The presence of structural and compositional inhomogeneities in the alloys was clearly seen.

**Table 2:** Co and W content of the CoW alloy at the points of interest using EDS analysis

	**Atomic percentage of Co**	**Atomic percentage of W**
Spectrum 1	68.25	31.75
Spectrum 2	47.80	52.20
Spectrum 3	46.40	53.60
Spectrum 4	49.33	50.67
Spectrum 5	64.64	35.36

## Conclusions

Investigations showed the presence of structural and compositional inhomogeneities in the CoW-CoNiW-NiW alloys. Atomic electron microscopy allowed us to determine the preferential areas of the structural relaxation and crystallization processes. The most intensive nanocrystal growth occurs on free surfaces. Based on direct observation of the atoms' movements, it was determined that the diffusion coefficient is in the range of 0.9 to 1.7 × 10^–18^ m^2^/s, which was significantly higher than the volume diffusion coefficient for similar alloys. This can be explained by the prevalence of surface diffusion, which can exceed volume diffusion by three to five orders of magnitude [26-28]. It was found that local changes in the composition can reach 18 at.% for the CoW alloy and 4 at.% for the NiW alloy. In addition, tungsten is more homogeneously distributed than nickel or cobalt. This is associated with the higher mobility of nickel and cobalt atoms in the electrolyte. Thicker areas of the alloys are enriched by nickel, whereas the thinner ones have increased tungsten percentages. This data can be used to predict the thermal stability of the CoW-CoNiW-NiW alloys.

## Competing interests

The authors declare that they have no competing interests.

## Authors’ contributions

EVP carried out HRTEM studies and drafted manuscript. EBM carried out HAADF STEM studies, carried out *in situ* TEM experiments and corrected the manuscript draft. OVV carried out EELS chemical analysis and participated in *in situ* TEM experiments. ANF carried out image and video processing and participated in TEM studies. AVD carried out EDS chemical analysis and participated in TEM studies. BNG participated in the design of the study, performed diffusion studies and corrected the manuscript draft. VSP conceived of the study and participated in its design and coordination. SSG carried out alloys deposition. All authors read and approved the final manuscript.

## Authors’ information

EVP is an associate professor of computer systems department in School of Natural Sciences in Far Eastern Federal University. He has a Ph.D. in Physics and great experience in electron microscopy. His scientific interests are electron microscopy, physics of condensed matter, image processing, and high-performance computations on GPU. EBM is currently a Ph.D. student of School of Natural Sciences in Far Eastern Federal University. His Ph.D. project focuses on electron microscopy of amorphous and nanocrystalline metallic alloys and their structure changes under external impact. OVV is a Ph.D. student of School of Natural Sciences in Far Eastern Federal University. His Ph.D. project focuses on electron microscopy and electron tomography of structure inhomogeneities in amorphous metallic alloys.ANF holds a BS degree in Information Systems from Far Eastern Federal University. He is currently working toward a master's degree in Information Systems and Technologies at Far Eastern Federal University. He has interests and experience in image processing, computer simulation and electron microscopy. AVD holds a BS degree in Information Systems from Far Eastern Federal University. He is currently working toward a master's degree in Information Systems and Technologies at Far Eastern Federal University. He has interests and experience in multiscale modeling and development high-performance solutions. BNG is a full professor of Computer Systems Department in School of Natural Sciences in Far Eastern Federal University. He has many years of experience in electron microscopy image processing and modeling. VSP is a full professor of Computer Systems Department in School of Natural Sciences in Far Eastern Federal University and head of electron microscopy and image processing laboratory. His research activities started in 1970s and were focused on electron microscopy and physics of condensed matter. SSG is chief researcher of Scientific and Practical Centre of Material Science, Belarus National Academy. His scientific interests are microstructure studies, magnetic and mechanical properties of electrolytically deposited amorphous metal alloys. He has great experience in electrochemistry and experienced in obtaining alloys with specified functional characteristics.

## Supplementary Material

Additional file 1 Nanocrystal growing in the NiW alloy. Click here for file
